# Exploring Study Design Foibles in Randomized Controlled Trials on Convalescent Plasma in Hospitalized COVID-19 Patients

**DOI:** 10.3390/life14070792

**Published:** 2024-06-22

**Authors:** Massimo Franchini, Carlo Mengoli, Arturo Casadevall, Daniele Focosi

**Affiliations:** 1 Department of Hematology and Transfusion Medicine, Carlo Poma Hospital, 46100 Mantua, Italy; 2 Johns Hopkins Bloomberg School of Public Health, Department of Molecular Microbiology and Immunology, Baltimore, MD 21205, USA; acasade1@jhu.edu; 3 North-Western Tuscany Blood Bank, Pisa University Hospital, 56124 Pisa, Italy

**Keywords:** COVID-19, convalescent plasma, randomized controlled trials

## Abstract

**Background**: Sample size estimation is an essential step in the design of randomized controlled trials (RCTs) evaluating a treatment effect. Sample size is a critical variable in determining statistical significance and, thus, it significantly influences RCTs’ success or failure. During the COVID-19 pandemic, many RCTs tested the efficacy of COVID-19 convalescent plasma (CCP) in hospitalized patients but reported different efficacies, which could be attributed to, in addition to timing and dose, inadequate sample size estimates. **Methods**: To assess the sample size estimation in RCTs evaluating the effect of treatment with CCP in hospitalized COVID-19 patients, we searched the medical literature between January 2020 and March 2024 through PubMed and other electronic databases, extracting information on expected size effect, statistical power, significance level, and measured efficacy. **Results**: A total of 32 RCTs were identified. While power and significance level were highly consistent, heterogeneity in the expected size effect was relevant. Approximately one third of the RCTs did not reach the planned sample size for various reasons, with the most important one being slow patient recruitment during the pandemic’s peaks. RCTs with a primary outcome in favor of CCP treatment had a significant lower median absolute difference in the expected size effect than unfavorable RCTs (20.0% versus 33.9%, P = 0.04). **Conclusions**: The analyses of sample sizes in RCTs of CCP treatment in hospitalized COVID-19 patients reveal that many underestimated the number of participants needed because of excessively high expectations on efficacy, and thus, these studies had low statistical power. This, in combination with a lower-than-planned recruitment of cases and controls, could have further negatively influenced the primary outcomes of the RCTs.

## 1. Introduction

Similar to what happened in several previous infectious outbreaks, plasma collected from recovered subjects was the first antibody-based therapy used to fight the recent COVID-19 pandemic [1,2]. In the USA, COVID-19 convalescent plasma (CCP) was first deployed under a registry [3]. After an analysis of registry data had identified a signal of efficacy [4], the Food and Drug Administration (FDA) issued emergency use authorization, which led to CCP transfusions in more than 500,000 COVID-19 patients [5]. In addition, CCP was the most intensively studied anti-SARS-CoV-2 therapeutic agent in the COVID-19 pandemic, with nearly 50 randomized controlled trials (RCTs) focusing on CCP being published (Supplementary Table S1) [6,7,8,9,10,11,12,13,14,15,16,17,18,19,20,21,22,23,24,25,26,27,28,29,30,31,32,33,34,35,36,37,38,39,40,41,42,43,44,45,46,47,48,49,50,51,52]. Such trials have identified the correct place for CCP among therapies against COVID-19, and it is more effective in blocking viral replication and disease progression when transfused with a high concentration of anti-SARS-CoV-2 neutralizing antibodies (nAbs) at the early stage (i.e., within 5 days from symptom onset), particularly in seronegative immunocompromised patients [53,54,55]. While previous RCTs have consistently showed that early CCP administration in COVID-19 outpatients is effective in reducing disease progression and the risk of hospitalization, later studies on the in-hospital use of CCP have yielded mixed results [56,57]. Various underlying factors have been suggested to be contribute to the discrepancy of results, including differences in nAb titers, inpatient characteristics, and the timing of CCP transfusion [58]. Furthermore, differences in study design among RCTs conducted during the four-year pandemic period may have been another reason. In particular, heterogeneity in sample size estimation, which is a parameter of power analysis closely related to the treatment effect and is crucial for determining the success or failure of a trial, is likely to have played a critical role in suboptimal study designs [59,60].

In this study, we systematically investigated the sample size calculations of published RCTs evaluating CCP treatment in hospitalized COVID-19 patients.

## 2. Material and Methods

**Figure 1:**
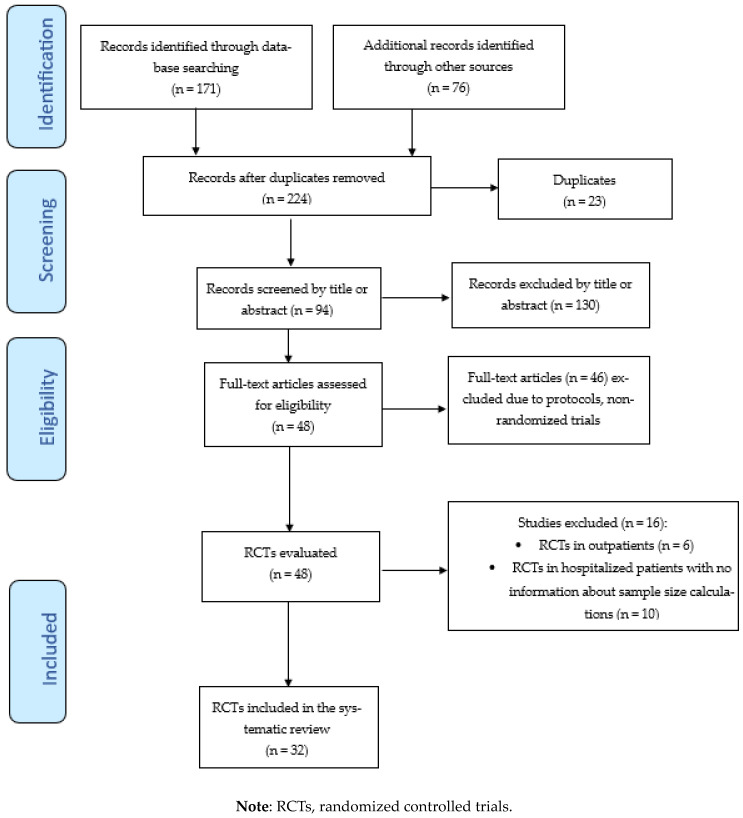
Flow chart of study inclusion process.

The aim of this systematic review was to evaluate sample size calculation and its possible influence on study results in RCTs conducted on using CCP treatment in patients hospitalized for COVID-19. A literature search of the PubMed (through Medline), EMBASE, Cochrane central, medRxiv, and bioRxiv databases was carried out between 1 January 2020 and 31 March 2024, using the English language as a filter. The Medical Subject Heading (MeSH) and search queries used were as follows: “(“COVID-19” OR “SARS-CoV-2” OR “coronavirus disease 2019”) AND (“convalescent plasma” OR “immune plasma” or “hyperimmune plasma”) AND (“randomized trial” OR “RCT”)”. We also screened the reference list of all the retrieved studies and review articles for additional studies not captured in our initial literature search. Finally, a PRISMA flowchart of the literature reviewing process was produced, and it is shown in Figure 1.

Only RCTs that enrolled patients hospitalized with COVID-19 of any disease severity and treated with CCP were included in this systematic review. The exclusion criteria were outpatient setting and the absence of a sample size calculation (sample size estimation was retrieved from the ‘Method’ section of each published study and/or from the study design of the registered protocol). The CCP treatment (intervention) was compared with any controls (i.e., standard treatment, placebo, CCP, or standard plasma). The following parameters were extracted from each study (Table 1 and Supplementary Table S1): study design, date of initial recruitment of patients, sample size estimation (expected size effect, statistical power, and significance level), number of expected and number of actually enrolled cases and controls, early termination of the study (and cause of study termination), primary outcome, and 28-day mortality rate (when present, reported as primary or secondary) in the CCP and control arms. When possible, the expected size effect was calculated as absolute or relative difference to render the results from different studies homogeneous and comparable. The articles underwent an independent evaluation for inclusion by two assessors (M.F. and D.F.), and disagreements were resolved by a third senior assessor (C.M.).

Within-trial Risk of Bias (ROB) was assessed using the Cochrane ROB tool for RCTs. The Cochrane ROB tool for RCTs addresses six specific domains: sequence generation, allocation concealment, blinding, incomplete data, selective outcome reporting, and other issues relating to bias [61]. The protocol was registered on PROSPERO (registration number: CRD42024537859).

Regarding statistical analysis, categorical variables were compared using a Chi-square test and presented as frequency and percentages, while continuous variables were compared with an independent *t*-test and paired *t*-test and presented as the mean ± standard deviation (SD). A *p* value less than 0.05 is considered statistically significant.

## 3. Results

A total of 247 studies were initially identified after querying electronic databases and manual searching. After the removal of 23 duplicates, we screened the titles and abstracts of 224 studies. After the exclusion of 130 records, 94 full-text articles were identified and assessed for eligibility, resulting in the selection of 48 RCTs. Finally, after the exclusion of 16 RCTs (see Supplementary Table S1 for the reasons for their exclusion), 32 RCTs [7,8,10,12,13,14,15,16,17,18,20,21,22,25,26,28,30,31,32,34,35,37,38,39,41,43,45,46,47,48,49,51] were included in the systematic review. The study selection process is summarized in the PRISMA flow diagram in Figure 1. The main characteristics of the studies included in the systematic review are summarized in Table 1. All studies included in this analysis involved patients hospitalized for COVID-19 of various degrees of severity, with the exception of two RCTs [16,47] which recruited critically ill patients admitted to an intensive care unit (ICU). All but two RCTs [31,49] began recruitment in 2020, during the first or second pandemic wave. In 10 of the 32 RCTs (31.2%), the number of cases/controls enrolled was lower than that planned by the study design. The reasons reported by the authors for early study termination were futility at interim analysis (three RCTs) [13,14,43], the EUA from FDA (one RCT) [10], the presence of high-titer nAbs in recipients at admission before CCP transfusion (one RCT) [15], vaccination coverage and the availability of anti-SARS-CoV-2 monoclonal antibodies (mAbs) (one RCT) [49], and slow recruitment due to the trial taking place during the interpandemic period (four RCTs) [21,26,31,32]. For 19 of the 32 (59.4%) selected RCTs, the primary outcome also included mortality rate, with the primary endpoint being reached in 6 studies (18.8%). The 28-day mortality rates differed widely among the studies, with the highest rate being recorded in the CONFIDENT [16] (35.4% in CCP group and 45.0% in control group) and COPLA-II [20] (53.2% in CCP group and 46.8% in control group) trials and the lowest rate being recorded in the ConPlas [17] (3.9% in CCP group and 8.2% in control group) and TSUNAMI [51] (6.1% in CCP group and 7.9% in control group) studies. Two studies [21,49] did not report deaths in either the treatment or control arm. Among the 28 RCTs reporting deaths as a primary or secondary outcome, two RCTs [16,34] (7.1%) reported a 28-day mortality rate significantly lower in CCP-treated patients than controls. Regarding sample size estimation, the great majority of RCTs were designed with a statistical power of 80% and a 5% level of significance. Wide inter-study variation in the expected relative difference in the primary outcome between the CCP treatment and control groups was observed, ranging from 25% to 50%. This heterogeneity among the RCTs was also evident when the expected absolute improvement in the primary outcome of CCP-treated patients was considered, ranging from 15% to 50%.

A statistically significant low in the median expected absolute difference was observed in studies with a favorable outcome [21,28] compared to those with an unfavorable outcome [7,12,15,18,22,38,39,45,46] (20.0% versus 33.9%, *p* = 0.04).

Regarding ROB analysis in included studies, we assessed seven studies with a low risk of bias in all the items considered (see Supplementary Figure S2). The remaining 25 studies were judged to have a high or unclear risk of bias for one or more domains. Nearly 70% of studies were open-label studies; hence, they were at risk of performance or detection bias (in studies with unmasked evaluators).

## 4. Discussion

Since the publication of the first RCTs on the use of CCP, it has been evident that the clinical effect of CCP depends on several factors, with the most important one being the phase of the viral infection (the earlier the plasma is transfused, the more effective the CCP treatment is) and its content in nAbs (the more nAbs there are in the CCP, the more effective it is). The latter factor matches the serologic status of CCP recipients: patients with a reduced or absent antibody response against SARS-CoV-2, such as immunocompromised patients, respond better to high-titer CCP therapy [55]. In addition to the timing and dosing of CCP, there are other key determining factors of CCP effectiveness, among which the study design must be mentioned, in particular the sample size estimation [62,63]. Sample size calculation is an essential component of a study protocol. The ex ante determination of the minimum number of observations that have to be recorded is essential in order to detect a supposed treatment effect, and thus, it is closely related to the success or the failure of the trial [64]. In turn, the calculation of the sample size of a new trial depends on the expected effectiveness of the treatment compared to the control. The greater the difference observed, the smaller the number of events to be collected. In other words, the sample size needed to assess the treatment effectiveness is higher when the real treatment effect is lower (Supplementary Table S2 and Supplementary Figure S1). Generally, RCTs with a small sample size are easy to conduct and economically sustainable, particularly for independent, non-sponsored trials, and, thus, they are usually preferred over those with a very large sample size, which are expensive and time consuming. By contrast, small-size RCTs are prone to having low statistical power and promote misleading inferences, while large-size RCTs are generally far more efficient in producing consistent evidence [65]. The a priori estimation of a given difference in the efficacy between an intervention and comparator during the planning of a study design is usually based on previous trials on the same topic, but, unfortunately, this is not possible for a new disease. As COVID-19 was a new illness, the investigators could not design RCTs using prior experience (the majority of the RCTs started concomitantly in 2020 during the first or second pandemic wave), and thus, they utilized results regarding CCP efficacy from trials conducted in previous coronavirus epidemics, such as the SARS and MERS epidemics, or results from uncontrolled SARS-CoV-2 studies. Thus, as shown by the analyses of the sample sizes of 32 RCTs, many estimated a 30 to 50% a priori relative reduction (or improvement) in the primary outcome by the intervention (CCP) to calculate the number of hospitalized COVID-19 patients to enroll. This approach was, however, wrong for at least two reasons: first, it became evident immediately after the outbreak of the pandemic that the COVID-19 pandemic had a different degree of severity compared to the two previous coronavirus epidemics. Furthermore, studies conducted (and published) in the early phase of the COVID-19 pandemic clearly showed that a “Lazarus effect” was not possible with either CCP or other antibody-based or small-molecule antivirals, meaning that a drug could be considered effective if it led to an improvement in the primary outcome in a range between 10% and 20% [57]. In fact, the closest historical account of the use of CCP in a pandemic was the use of convalescent serum in the 1918 influenza pandemic, where a favorable size effect of 20% was estimated from retrospective analysis [66]. A posteriori, this was also confirmed to be true for CCP, as documented by the most recent literature review, which showed an overall 13% reduced risk of mortality of CCP compared with standard-of-care treatment or placebo in hospitalized COVID-19 patients [56]. This issue was further complicated by the wide heterogeneity in the methodologies used by the different RCTs for sample size estimation (relative or absolute difference and odds ratios), hampering among-study comparisons.

In addition to this methodological heterogeneity, COVID-19 presented challenges that are not usually encountered in RCTs, including the fact that the efficacy of antibody therapy varied with length of illness and the rapid progression of the underlying disease. For example, the efficacy of CCP in preventing the progression of disease to hospitalization exceeds that of monoclonal antibodies when given in the first 5 days of infection [54], but CCP has little or no efficacy when administered after the third day of hospitalization [4], whereas the overall survival benefit associated with CCP, considering over thirty RCTs, was 13%, and the estimated efficacy in reducing mortality when administered in the first three days of hospitalization using high-titer plasma was 37% [56]. Given that the efficacy of CCP diminishes rapidly with time, the inevitable delays associated with enrollment, randomization, and CCP administration in RCTs served to further reduce the likelihood of finding a favorable effect. Hence, the rapid progression of COVID-19, combined with the reduced efficacy of CCP as a function of time, significantly increased the heterogeneity of patients enrolled in RCTs, which further reduced the likelihood of finding statistically significant effects given the sample sizes studied.

Approximately one third of the RCTs evaluated in the present systematic review were terminated prematurely for a series of reasons, but no company-sponsored RCTs of antibody-based or small molecule antivirals were terminated prematurely. Among the reasons for premature termination, the most relevant reason is problems regarding patient enrolment between the pandemic waves or because of the EUA of CCP granted by the FDA, both of which deprived the US-based RCTs on CCP of many patients. Despite this, it is noteworthy that at least two RCTs including hospitalized patients were completed in the USA [18,36], showing that it was possible to test CCP under EUA. It is noteworthy that many CCP RCTs did not have commercial sponsors and, thus, in terms of patient recruitment, they did not have the financial incentives that are often associated with pharmaceutical trials. Likewise, the cessation of some RCTs at interim analysis for futility could have been due to the vicious circle created by the discrepancy between the virtual (expected) and the real (observed) treatment effect. Adding to these hurdles, the enrolment of patients in CCP trials often made them ineligible to participate in other RCTs for other COVID-19 therapies supported by pharmaceutical companies that usually provide payment for each participant, thus creating further disincentives for continued recruitment in CCP RCTs during the highs and lows of the SARS-CoV-2 waves.

Therefore, such RCTs could not have sufficient statistical power for two reasons, i.e., reduced enrolment in the context of an already reduced sample size that had been calculated by overestimating the CCP treatment effect. In support of the latter argument, we observed that the expected absolute difference between intervention and comparator in the primary outcome was significantly lower in favorable versus unfavorable studies. Furthermore, it is noteworthy that an industry-led double-blinded phase 3 RCT testing the efficacy of mAb tixagevimab–cilgavimab versus placebo in hospitalized COVID-19 patients showed benefits for the mAb combination, in which the design assumed a 20% effect (power 90%, 5% significance level) for sample size estimation, and the expected recruitment target was fully achieved [67].

In conclusion, the results of our systematic review, which was performed on 32 RCTs, clearly underline the important role of sample size calculation in the design of different studies evaluating CCP efficacy in hospitalized COVID-19 patients. From our systematic analysis of the literature on this topic, we have found that the sample size estimate is a key determinant of treatment effectiveness. Indeed, it is a fact that most RCTs lack statistical power due to an overlooked size effect that could negatively influence their results and restrict them from reaching enough events, in both cases and controls, to correctly evaluate the effect of CCP treatment. But what would have been the results of these studies if they had set their size effect at a lower (below 20%), and more realistic, difference level? At this time, it is not possible (and not methodologically correct) to try to answer to this question ex post. Perhaps, however, if this had happened, the history of the results of many RCTs and of the clinical use of CCP worldwide during the pandemic would have turned out differently.

## Figures and Tables

**Table 1:** Characteristics of the 32 RCTs included in the analysis.

Study, Year [ref.] NCT	Study Design	Recruitment Start (mm/dd/yy)	Sample Size Estimation			Cases/Controls		Early Termination (Reason)	Primary Outcome		28-Day Mortality
			Effect Size ^1^	Power	CL	Expected	Actual		Endpoint	Reached	
Balcells, 2021 [7]	Open-label RCT including hospitalized COVID-19 patients	05/10/2020	AD: 35%	80%	95%	29 early CCP + ST/29 late CCP + ST	28 early CCP + ST/30 late CCP + ST	No	Composite of mechanical ventilation; hospitalization for >14 days or death	No	Early CCP: 5/28 (17.9%) Late CCP: 2/30 (6.7%) P = 0.25
Baldeon, 2021 [8]	Double-blind, placebo-controlled RCT including hospitalized pts. with moderate COVID-19	05/01/2020	RD: 40%	80%	95%	100 CCP/95 SP	63 CCP/95 SP	No	Survival rate at 28 days	No	CCP: 11.1% SP: 12.6% P = NS
Bennett-Guerrero, 2021 [10]	Double-blind RCT including hospitalized COVID-19 patients	04/08/2020	RD: 25%	90%	95%	400 CCP/100 SP	59 CCP/15 SP	Yes (EUA from FDA)	Number of ventilator-free days from randomization to day 28	No	CCP: 14/59 (23.7%) SP: 4/15 P = 0.80 (26.7%)
CAPSID, 2021 [12] NCT04433910	Open-label RCT including hospitalized patients with severe COVID-19	08/30/2020	AD: 30%	80%	95%	48 CCP + ST/48 ST	53 CCP + ST/52 ST	No	Composite outcome of survival and no longer fulfilling criteria for severe COVID-19 on day 21	No	CCP + ST: 8/53 (15.1%) ST: 14/52 (26.9%) P = 0.16
CCAP-2, 2022 [13] NCT04345289	Double-blind, placebo-controlled RCT including hospitalized COVID-19 pts.	06/13/2020	RD: 35%	80%	95%	353 CCP/177 PL	98 CCP/46 PL	Yes (futility)	Clinical status on day 14	No	CCP: 12/98 (12.2%) PL: 3/46 (6.5%) P = NS
CONCOR-1, 2021 [14] NCT04348656	Open-label RCT including hospitalized patients with COVID-19	05/14/2020	RD: 25%	80%	95%	800 CCP/400 ST	625 CCP/313 ST	Yes (futility)	Composite outcome of intubation or death by day 30	No	CCP: 114/548 (20.8%) ST: 62/303 (20.5%) P = 0.90
ConCOVID, 2021 [15]	Open-label, RCT including hospitalized patients with severe COVID-19	04/08/2020	AD: 50%	80%	95%	213 CCP/213 ST	43 CCP/43 ST	Yes (nAbs in recipients at admission)	Overall mortality until discharge	No	CCP: 6/43 (13.9%) ST: 11/43 (25.6%) P = NS
CONFIDENT, 2023 [16] NCT04558476	Open-label RCT including mechanically ventilated COVID-19 patients	09/01/2020	RD: 33%	80%	95%	250 CCP/250 ST	237 CCP/238 ST	No	28-day mortality	Yes	CCP: 84/237 (35.4%) ST: 107/238 (45.0%) P = 0.03
ConPlas, 2021 [17] NCT04345523	Open-label RCT including hospitalized patients with COVID-19	04/04/2020	RD: 50%	80%	95%	175 CCP/175 ST	179 CP/171 ST	No	Clinical worsening at 14 days	Yes	CCP: 7/179 (3.9%) ST: 14/171 (8.2%) P = 0.11
CONTAIN COVID-19, [18] NCT04364737	Open-label RCT including hospitalized patients with COVID-19	04/17/2020	AD 18%	80%	95%	463 CCP/463 PL	468 CCP/473 PL	No	Clinical status on day 14	No	CCP: 59/462 (12.8%) PL: 71/462 (15.4%)
COPLA-II, 2022 [21] NCT04425915	Open-label, phase III RCT including severe COVID-19 patients	06/14/2020	RD: 25%	80%	95%	190 CCP + ST/190 ST	200 CCP + ST/200 ST	No	Clinical improvement measured by a two-point reduction in the ordinal scale	Yes	CCP + ST: 42/200 (53.2%) ST: 37/200 (46.8%) P = 0.62
Coplasma-2020, 2022 [22]	RCT including hospitalized patients with COVID-19	07/01/2020	AD: 20%	80%	95%	126 CCP/63 ST	37 CCP/17 ST	Yes (slow recruitment)	Time for clinical improvement within 21 days	Yes	CCP: 0/37 ST: 0/17
CORIPLASM, 2023 [23] NCT04345991	Open-label RCT including hospitalized COVID-19 patients	04/16/2020	AD: 30%	97.2%	NR	60 CCP/60 ST	60 CCP/60 ST	No	WHO score > 6 on day 4 and survival on day 14	No	CCP: 7/60 (11.7%) ST: 12/60 (20.0%) P = NS
DAWn-plasma, 2021 [26]	RCT including patients hospitalized for COVID-19	NR	RD: 50%	80%	95%	322 CCP/161 ST	320 CCP/163 ST	No	Patient alive without mechanical ventilation at day 15	No	CCP: 8.8% ST: 8.8%
Denkinger, 2023 [27]	Open-label RCT including cancer patients hospitalized with severe COVID-19	09/03/2020	RD: 35%	80%	95%	87 CCP + ST/87 ST	68 CCP + ST/66 ST	Yes (slow recruitment)	Time to clinical improvement of two points on a 7-point ordinal scale	Yes	CCP + ST: 14/68 (20.6%) ST: 19/66 (28.8%) P = NS
Hamdy Salman, 2020 [29]	RCT including hospitalized COVID-19 pts.	06/01/2020	AD: 20%	80%	95%	15 CCP + ST/15 ST	15 CCP + ST/15 ST	No	Reduction in two or more of a four-category illness severity scale over 5 days	Yes	NR
Kirenga, 2021 [31] NCT04542941	RCT including hospitalized COVID-19 patients	09/21/2020	RD: 40%	80%	95%	66 CCP + ST/66 ST	69 CCP + ST/67 ST	No	Viral clearance by day 28	No	CCP + ST: 10/69 (14.5%) ST: 8/67 (11.9%) P = 0.66
Khawaja, 2024 [32]	RCT including hospitalized COVID-19 pts.	02/02/2021	RD: 45%	80%	95%	130 HT-CCP/130 LT-CCP/130 PL	18 HT-CCP/19 LT-CCP/20 PL	Yes (slow recruitment)	Reduction in intubation and corticosteroid support, safety.	No	HT-CCP: 0/18 LT-CCP: 1/19 (5.2) PL: 0/20
Li, 2020 [33] ChiCTR2000029757	Open-label RCT including patients with severe or life-threatening COVID-19	02/14/2020	RD: 40%	80%	95%	100 CCP + ST/100 ST	52 CCP + ST/51 ST	Yes (slow recruitment)	Time to clinical improvement within 28 days	No	CCP + ST: 8/51 (15.7%) ST: 12/50 (24.0%) P = 0.30
O’Donnell, 2021 [35]	Double-blind RCT including hospitalized COVID-19 pts.	04/21/2020	OR: 1.5	82%	NR	146 CCP/73 SP	150 CCP/73 SP	No	Clinical status at 28 days	No	CCP 19/150 SP 18/73 P = 0.03
PassITON, 2022 [36] NCT04362176	Double-blind, placebo-controlled RCT including hospitalized COVID-19 pts.	04/28/2020	OR: ≤0.73	80%	95%	500 CCP/500 PL	487 CCP/473 PL	No	Clinical status at 14 days	No	CCP: 89/482 (18.5%) PL: 80/465 (17.2%) P = NS
PLACID, 2020 [38] CTRI/2020/04/024775	Open-label, parallel-arm, phase II RCT including hospitalized patients with moderate COVID-19	04/22/2020	RD: 50%	80%	95%	226 CCP + ST/226 ST	235 CCP + ST/229 ST	No	Composite outcome of progression to sever disease or all-cause mortality at 28 days	No	CCP + ST: 44/235 (18.7%) ST: 41/229 (17.9%) P = NS
PLACO COVID, 2022 [39] NCT04428021	Three-arm blinded RCT on hospitalized COVID-19 patients; power 80%	06/01/2020	AD: 15%	80%	95%	60 CCP + ST/60 ST/60 SP + ST	60 CCP + ST/60 ST/60 SP + ST	No	30-day mortality	No	CCP + ST: 14/60 (23.3%) ST: 12/60 (20.0%) P = 0.69
PLACOVID, 2022 [40] NCT04547660	Open-label RCT including hospitalized COVID-19 patients	07/15/2020	AD: 20%	80%	95%	80 CCP + ST/80 ST	80 CCP + ST/80 ST	No	Proportion of patients with clinical improvement at day + 28	No	CCP + ST: 18/80 (22.5%) ST: 13/80 (16.3%) P = 0.32
PlasmAr, 2021 [42] NCT04383535	Double-blind, placebo-controlled RCT including severe COVID-19 patients	05/28/2020	OR: 1.8	80%	95%	222 CCP/111 PL	228 CCP/105 PL	No	Patient’s clinical status at 30 days	No	CCP: 25/228 (11.0%) PL: 12/105 (11.4%) P = NS
PROTECT, 2022 [44] NCT04516811	Double-blind, phase III RCT including patients with moderate to severe COVID-19	09/30/2020	RD: 33%	80%	95%	300 CCP/300 PL	52 CCP/51 PL	Yes (futility)	Clinical improvement at day 28	No	CCP: 11/52 (21.5%) PL: 13/51 (25.5%) P = NS
Ray, 2022 [46] CTRI/2020/05/025209	Open-label, phase II RCT including hospitalized COVID-19 patients	05/31/2020	AD: 23%	NR	NR	40 CCP/40 ST	40 CCP/40 ST	No	All-cause mortality by day 30	No	CCP: 10/40 (25.0%) ST: 14/40 (35.0%) P = NS
RECOVERY, 2021 [47] NCT04381936	Open-label RCT including hospitalized COVID-19 patients	05/28/2020	AD: 20%	90%	99%	5500 CCP + ST/550 ST	5795 CCP + ST/5763 ST	No	28-day mortality	No	CCP + ST: 1399/5795 (24.1%) ST: 1408/5763 (24.4%) P = 0.95
REMAP-CAP, 2021 [48] NCT02735707	Open-label RCT including critically ill COVID-19 pts.	03/09/2020	OR: 1.2	NR	NR	NR	1075 CCP/904 PL	No	Median number of organ support-free days	No	CCP: 352/1074 (32.8%) PL: 300/904 (33.2%) P = NS
Rojas, 2022 [49] NCT04332835	Single-blinded, parallel-controlled RCT in including hospitalized COVID-19 patients	08/08/2020	RD: 30%	90%	95%	46 CCP + ST/46 ST	40 CCP + ST/43 ST	No	Reduction in viral load at day 28	No	CCP + ST: 6/46 (13.0%) ST: 2/45 (4.4%) P = 0.15
Saito, 2023 [50]	Open-label RCT including hospitalized patients with mild COVID-19	02/24/2021	RD: 50%	90%	95%	96 CCP/96 ST	10 CCP/11 ST	Yes (vaccination coverage and mAB availability)	Time-weighted average change in the SARS-CoV-2 viral load	No	No deaths recorded
TSUNAMI, 2021 [52] NCT04716556	Open-label RCT including hospitalized COVID-19 patients	07/15/2020	RD:40%	80%	95%	237 CCP + ST/237 ST	231 CCP + ST/239 ST	No	Composite of worsening respiratory failure or death < 30 days	No	CCP + ST: 14/231 (6.1%) ST: 19/239 (7.9%) P = 0.43

Abbreviations: CCP, COVID-19 convalescent plasma; SP, standard plasma; RD, relative difference; CL, confidence level; ST, standard therapy; PL, placebo; NR, not reported; WHO, World Health Organization; HT-CCP, high-titer CCP; ST-CCP, standard-titer CCP; LT-CCP, low-titer CCP; NCT, National Clinical Trial identifier number; NS, not significant; mAbs, monoclonal antibodies; EUA, emergency use authorization; FDA, Food and Drug Administration; nAbs, neutralizing antibodies. ^1^ The size effect was reported as absolute difference (AD), relative difference (RD), or as an odds ratio (OR) in the primary outcome between cases and controls.

## Data Availability

This manuscript generated no novel dataset.

## Supplementary Materials

The following supporting information can be downloaded at: https://www.mdpi.com/article/10.3390/life14070792/s1, Supplementary Table S1. Randomized controlled trials excluded from the analysis. Supplementary Table S2. Sample size estimation. Supplementary Figure S1. The non-linear relationship between expected effected size and necessary recruitment in each study arm. Supplementary Figure S2. Risk of Bias (ROB) analysis of the included studies. PRISMA checklist for this study. Ref. [68] is Supplementary Materials file.
